# Acetylation of Atp5f1c Mediates Cardiomyocyte Senescence via Metabolic Dysfunction in Radiation-Induced Heart Damage

**DOI:** 10.1155/2022/4155565

**Published:** 2022-09-15

**Authors:** Zhimin Zeng, Peng Xu, Yanqing He, Yali Yi, Zhicheng Liu, Jing Cai, Long Huang, Anwen Liu

**Affiliations:** ^1^Department of Oncology, The Second Affiliated Hospital of Nanchang University, Nanchang, Jiangxi Province, China; ^2^Jiangxi Key Laboratory of Clinical Translational Cancer Research, Nanchang, Jiangxi Province, China; ^3^Radiation Induced Heart Damage Institute of Nanchang University, Nanchang, Jiangxi Province, China; ^4^Department of Hospital Infection Management, The Second Affiliated Hospital of Nanchang University, Nanchang, Jiangxi Province, China; ^5^The first Clinical College of Nanchang University, Nanchang, Jiangxi Province, China

## Abstract

**Objective:**

Ionizing radiation (IR) causes cardiac senescence, which eventually manifests as radiation-induced heart damage (RIHD). This study is aimed at exploring the mechanisms underlying IR-induced senescence using acetylation proteomics.

**Methods:**

Irradiated mouse hearts and H9C2 cells were harvested for senescence detection. Acetylation proteomics was used to investigate alterations in lysine acetylation. Atp5f1c acetylation after IR was verified using coimmunoprecipitation (Co-IP). Atp5f1c lysine 55 site acetylation (Atp5f1c K55-Ac) point mutation plasmids were used to evaluate the influence of Atp5f1c K55-Ac on energy metabolism and cellular senescence. Deacetylation inhibitors, plasmids, and siRNA transfection were used to determine the mechanism of Atp5f1c K55-Ac regulation.

**Results:**

The mice showed cardiomyocyte and cardiac aging phenotypes after IR. We identified 90 lysine acetylation sites from 70 protein alterations in the heart in response to IR. Hyperacetylated proteins are primarily involved in energy metabolism. Among them, Atp5f1c was hyperacetylated, as confirmed by Co-IP. Atp5f1c K55-Ac decreased ATP enzyme activity and synthesis. Atp5f1c K55 acetylation induced cardiomyocyte senescence, and Sirt4 and Sirt5 regulated Atp5f1c K55 deacetylation.

**Conclusion:**

Our findings reveal a mechanism of RIHD through which Atp5f1c K55-Ac leads to cardiac aging and Sirt4 or Sirt5 modulates Atp5f1c acetylation. Therefore, the regulation of Atp5f1c K55-Ac might be a potential target for the treatment of RIHD.

## 1. Introduction

Radiotherapy is the mainstay of treatment for thoracic cancer (e.g., thymoma and cancers of the lung, breast, and esophagus) and lymphoma [1]. More than half of the patients receive radical or palliative radiotherapy during anticancer treatment [2]. Large clinical studies have found that the long-term survival of patients who have undergone thoracic radiotherapy is impaired by radiation-induced heart damage (RIHD) [3–5]. RIHD is mainly observed many years after patients receive thoracic radiotherapy, manifesting as coronary artery disease, ischemic heart disease, pericarditis, conduction defects, and valvular dysfunction [6–8]. RIHD can impact prognosis and increase cardiac mortality and has become a challenge in clinical practice [9, 10].

Cellular senescence is a risk factor for cardiovascular disease and is correlated with cardiac dysfunction [11]. Senescence is a permanent state of cell cycle arrest that promotes tissue remodeling and often occurs in different physiological and pathological processes [12]. Cardiomyocyte senescence can impair metabolic and contractile dysfunction and activate fibroblasts [13]. The mechanism of RIHD is known to generate oxidative stress, damage DNA, injure endothelial cells, and secrete cytokines, which may cause metabolic disorders and nucleus damage, leading to cell apoptosis or necrosis [14, 15]; oxidative stress, DNA damage, and continuous inflammation have also been implicated in the mechanism of cardiomyocyte senescence [16]. Moreover, various pathogenic factors can cause cardiac and cardiomyocyte senescence [17]. Cardiac senescence can be characterized by systolic dysfunction and contribute to the initiation of heart failure [18]. Survivors of thoracic malignancies who receive radiotherapy may present with cardiac disease with or without deterioration of myocardial contractility after decades [19, 20]. Ionizing radiation- (IR-) induced DNA damage, senescence-associated inflammatory factors, and ROS are also hallmarks of cardiomyocyte senescence [21]. Senescence and the DNA damage-associated >BRCA1 and p53/p21 signaling axis regulated by IR can cause cardiovascular injury, which eventually leads to RIHD [22, 23]. Cardiac cellular senescence can also manifest as inflammation- or ROS-induced telomere shortening and senescence-associated secretory phenotypes (SASPs) [16, 24]. Thus, IR might cause cardiac senescence. However, the molecular mechanisms underlying radiation-induced senescence remain to be elucidated. Epigenetic regulation is closely related to cardiac senescence [13]. Lysine acetylation of proteins is a conserved posttranslational modification that modifies protein structure and integrates biological processes such as metabolism, circadian rhythm, and gene transcription in organisms [25, 26]. The molecular mechanisms underlying acetylated protein alterations have been studied in many cardiac diseases [27]. Certain modifications, such as acetylation, are generally associated with cardiac hypertrophy or heart failure [28, 29]. Protein acetylation is regulated by lysine acetyltransferases and lysine deacetylases [30]. Some lysine acetyltransferases and lysine deacetylases regulate energy metabolism and have been intensively studied in mammals [25]. Sirtuins (Sirts) are a family of nicotinamide adenine dinucleotide- (NAD+-) dependent deacetylases with versatile functions [31], especially in metabolism and aging [32]. In addition, the acetylation level of proteins is especially relevant in some metabolic diseases, including atherosclerosis, diabetes mellitus, and polycystic ovary syndrome [33, 34]. IR can cause various posttranslational modifications, and epigenetic regulation plays a central role in normal tissue injury induced by radiation exposure [35, 36]. Apoe-/- mice that received 6 Gy total body irradiation presented an increase in cardiac mitochondrial protein acetylation levels and metabolic damage [37]. However, the relationship between acetylation alteration and radiation-induced cardiac senescence remains unclear.

In our previous study, we found that high-energy X-rays resulted in cardiometabolic disorders, collagen deposition, and suppressed cardiac function [38]. In this study, we aimed to investigate the mechanisms of radiation-induced cellular senescence, mainly using tandem mass tags- (TMT-) labeled acetylation proteomics.

## 2. Methods

### 2.1. Establishment of Mouse RIHD Model

The mouse RIHD model and radiation parameters were established according to our previous study [38]. The hearts of 10 experimental C57BL/6 male mice (Shanghai Institute of Biochemistry and Cell Biology, China) aged 8 weeks were irradiated with a 16 Gy/1 fraction and killed at 1 month (5 mice) and 5 months (5 mice) after irradiation. At 5 months, mice in the control group (13 mice) were euthanized, and heart tissues were harvested for further study. All mice were euthanized after intraperitoneal injection of pentobarbital sodium. The mice were bred and housed with adequate water and food. The experiments were approved by the Animal Ethics Committee of the Second Affiliated Hospital of Nanchang University.

### 2.2. Cell Culture

H9C2 cells were cultured at 37C with 5% CO_2_ in high-glucose Dulbecco's Modified Eagle Medium (Gibco, USA) supplemented with 10% fetal bovine serum (FBS) (Gibco, USA), 100U/mL penicillin, and 50*μ*g/mL streptomycin (Solarbio, China) in 10cm culture dishes (Nest, China). The cell lines were divided into an irradiation group (Varian Clinic 23EX, USA) and a control group (0Gy). The irradiated dishes were covered with a 1cm bolus and received a single dose of 10Gy at a dose rate of 600cGy/min, and source-to-surface distance (SSD) was 100 cm. The cells were harvested after 12, 24, and 48h of irradiation. Additionally, control cells were harvested after 48h. H9C2 cells were more energetically similar to primary cardiomyocytes, especially in energy metabolism patterns, compared with HL-1 cells [39].

### 2.3. Plasmid Construction and Transfection

Plasmids Atp5f1c K55Q-6his, Atp5f1c K55R-6his, and Atp5f1c-6his were constructed by Shanghai Jikai Gene Chemical Technology Co., Ltd. (Shanghai, China), and cDNA fragments were cloned into GV417 eukaryotic expression vectors. The resulting PCR products were digested using NheI and BamHI. Successful mutations were confirmed by DNA sequencing. Primers for the plasmid encoding the Atp5f1c mutant K55Q, K55R, and WT were generated and are listed in Supplementary Table 1.

Overexpression plasmids of HA-Sirt3, HA-Sirt4, and HA-Sirt5 were constructed by Shanghai Jikai Gene Chemical Technology Co., Ltd., and cDNA fragments were cloned into GV366 eukaryotic expression vectors. The resulting PCR product was digested using BamHI and XhoI. Successful mutations were confirmed by DNA sequencing. Primers for the plasmid encoding Sirt3 overexpression are listed in Supplementary Table 2. Plasmids were transiently transfected into cells using Lipo3000 (Invitrogen, Waltham, Massachusetts, USA) according to the manufacturer's instructions. After transfection, the cells were harvested after 48 h and subjected to further analysis.

### 2.4. RNA Interference

siRNAs of Sirt3, Sirt4, and Sirt5 were synthesized by Han Heng Biotechnology (Shanghai, China) Co., Ltd., and the oligonucleotide sequences are listed in Supplementary Table 3. siRNAs were transfected into H9C2 cells using Lipo3000 (Invitrogen, USA) according to the manufacturer's protocol. The efficiency of the gene knockdown was verified using qPCR.

### 2.5. qPCR

Total RNA was extracted from the heart apex using TRIzol Invitrogen (Solarbio, Beijing, China) following the manufacturer's instructions and then reverse-transcribed into cDNA using the PrimeScript™ RT reagent Kit with gDNA Eraser (TaKaRa, Japan). PCR amplifications were performed using TB Green Premix Ex Taq™ (TaKaRa, Japan), following the manufacturer's instructions. Actin and GAPDH served as controls. The primer sequences are listed in Supplementary Tables 4 and 5.

### 2.6. Western Blotting

Total proteins from the animal heart apex or H9C2 cells were extracted using RIPA (Solarbio, Beijing, China), and protein concentration and western blotting were performed as previously described [40] The primary antibodies used in this study were anti-acetyllysine rabbit pAb (1 : 1000; Jingjie PTM BioLabs, Inc., China), anti-Atp5f1 monoclonal antibody (1 : 1000; Proteintech, Wuhan, China), anti-p21 monoclonal antibody (1 : 1000; Boster, Wuhan, China), HA-tagged monoclonal antibody (1 : 20000; Proteintech, Wuhan, China), 6× His-tagged monoclonal antibody (1 : 10000; Proteintech, Wuhan, China), GAPDH monoclonal antibody (1 : 20000; Proteintech, Wuhan, China), and anti-*α*-tubulin monoclonal antibody (1 : 1000; Boster). Anti-rabbit (1 : 10000) and anti-mouse (1 : 10000) antibodies were purchased from Proteintech.

### 2.7. Coimmunoprecipitation (Co-IP)

Briefly, samples were extracted from control and irradiated H9C2 cells (10 cm dish per sample) using NP-40 (Beyotime, China). Co-IP experiments were performed using SureBeads Protein A/G Magnetic Beads (Bio-Rad, CA, USA). Briefly, 30 *μ*L of SureBeads protein A and G was magnetized and washed with 0.1% PBS/Tween 20 (PBST) and then incubated with 2 *μ*g anti-acetyllysine rabbit pAb (Jingjie PTM BioLabs Inc., Hangzhou, China) or IgG rabbit antibody (Abcam, USA) at room temperature for 30 min. Next, the incubated beads were washed five times with 0.1% PBST and then incubated with 100 *μ*g of extracted protein overnight at 4°C. Proteins were electrophoresed on SDS-PAGE gels and immunoblotted on a PVDF membrane (Millipore, USA). Membranes were incubated with Atp5f1c rabbit antibodies (1 : 1000; Proteintech, Wuhan, China) and detected using a peroxidase-conjugated secondary antibody (1 : 20000; Abcam, USA) with ECL Blotting Substrates (Beyotime, China). Membranes were visualized by chemiluminescence (Bio-Rad, USA) and quantified using the ImageJ 14.9 software (ImageJ, Marlyand, USA).

### 2.8. Proteomics of Lysine Acetylation

Quantitative proteomic analysis through TMT was performed by Jingjie PTM BioLab (Hangzhou, China) Co., Ltd. The sham-irradiated and 5-month-irradiated heart apexes were ground in liquid nitrogen and lysed in buffer (3 *μ*M trichostatin A, 8 M urea, and 1% protease inhibitor). The remaining precipitate was removed by centrifugation at 12,000 × *g* for 10 min at 4°C. The subsequent procedures (trypsin digestion, TMT labeling of peptides, HPLC fractionation, and LC-MS/MS analysis) were performed as previously described [41]. The resulting MS/MS data were analyzed using the MaxQuant search engine (v.1.5.2.8) at Jingjie PTM BioLab. The database search method is shown in the Supplementary Material (Data S1).

### 2.9. Bioinformatics Analysis

#### 2.9.1. Functional Classification and Subcellular Localization Analysis

GO annotation of the proteome was derived from the UniProt-GOA database (https://www.ebi.ac.uk/GOA/). The WoLFSPORT database was used to predict the subcellular localization of proteins (https://www.genscript.com/psort/wolf_psort.html).

#### 2.9.2. Functional Enrichment

GO annotations can be divided into three categories: biological processes, cellular components, and molecular functions. Two-tailed Fisher's exact test was used to assess the enrichment of differentially expressed proteins (DEPs) against all identified proteins. Kyoto Encyclopedia of Genes and Genomes (KEGG) enrichment analysis was performed at https://www.genome.jp/kegg/.

#### 2.9.3. Protein-Protein Interaction Network

All differentially expressed modified protein database accessions or sequences were searched against the STRING database (version 11.0; https://www.string-db.org/) for protein-protein interactions. Only interactions between proteins belonging to the searched dataset were selected, thereby excluding external candidates. STRING defines a metric called the “confidence score” to define interaction confidence; we fetched all interactions that had a confidence score > 0.7 (high confidence). Interaction networks from STRING were visualized using Cytoscape 3.7.2. Additionally, CytoHubba was used to study essential nodes in the network with 11 methods (DMNC and degree exhibit a satisfactory comparative performance), completed to explore hub genes [42].

### 2.10. Telomere Length Measurement

The relative average telomere length was examined using a q-PCR-based telomere assay described previously [43]. The Ct values of telomeres (*T*) and the single copy gene 36b4 (*S*) were used as reference genes and were determined by qPCR. The ratio of telomere (*T*) repetitive copy number to single copy internal reference gene (*S*) can be used to assess the relative telomere length (*T*/*S*), whereas the *T*/*S* ratio is proportional to telomere length. The calculation formula of *T*/*S* is as follows: *T*/*S* = [2 CT (telomeres)/2 CT (single copy gene)] = 2 − ΔCT. The primers used were as follow: telomere-F primer (GGTTTTTGAGGGTGAGGGTGAGGGTGAGGGTGAGGGT), telomere-R primer (TCCCGACTATCCCTATCCCTATCCCTATCCCTATCCCTA), mouse 36B4-F primer (ACTGGTCTAGGACCCGAGAAG), and mouse 36B4-R primer (TCAATGGTGCCTCTGGAGATT). Telomere PCR was performed at 95°C for 10 min followed by amplification rounds consisting of 40 cycles at 95°C for 15 s, 60°C for 1 min, and 72°C for 30 s. The telomere repeat copy number to single gene copy number (*T*/*S*) ratio was determined using a Bio-Rad connection in a 96-well format.

### 2.11. ATP Synthase Activity Assay

Mitochondria were isolated using a Cell Mitochondria Isolation Kit (C3601; Beyotime, China). ATP synthase activity was measured using an ATP Synthase Enzyme Activity Microplate Assay Kit (ab109714; Abcam, USA). Briefly, samples of the transfected H9C2 cells were collected. ATP synthase from these samples was immunocaptured within the wells, and its enzyme activity was measured by determining the production of ADP, which is coupled with the oxidation of nicotinamide adenine dinucleotide hydrogen (NADH) to NAD^+^, and monitored as a decrease in absorbance at 340 nm in accordance with the manufacturer's protocol.

### 2.12. ATP Assay

ATP was measured using an ATP assay kit (Beyotime, China) according to the manufacturer's instructions. H9C2 cells were transfected into 6-well plates. Cell samples were added to 200 *μ*L of lysis buffer and centrifuged at 12,000 × g at 4°C for 5 min to obtain the supernatant. Supernatant samples were mixed with 100 *μ*L of ATP detection working buffer and measured using a multifunctional microplate reader (Thermo Scientific Microplate Reader, Varioskan LUX, Finland).

### 2.13. Senescence-Associated-Galactosidase Activity Assay

The transfected cells were isolated and cultured as described above. The cells were incubated with recombinant adiponectin or 25 mmol/L glucose for 72 h. Cellular senescence was detected using a senescent cell staining kit (Beyotime, China). Blue-stained and total cells were counted, and the percentage of galactosidase–positive cells was calculated.

### 2.14. Cardiac Echocardiogram

Transthoracic echocardiography was performed using the Vevo 2100 Ultrasound System (VisualSonics, Toronto, Canada) according to a previous study [40]. Two-dimensional guided M-mode echoes were obtained at the level of the largest left ventricle (LV). The left ventricular posterior wall at the end of diastole was measured using M-mode imaging. LV ejection fraction (EF) was calculated from the measured ventricular dimensions.

### 2.15. Statistical Analysis

All data are presented as the mean ± standard deviation (SD) of at least three replicates. Statistical differences between multiple comparisons were determined using one-way analysis of variance with least significant difference or Tukey *post hoc* test, and differences between two groups were determined using Student's *t*-test. Statistical analyses were performed with SPSS version 20 (IBM Corp.) and GraphPad Prism 8.0. *p* < 0.05 was considered to indicate a statistical significance.

## 3. Results

### 3.1. Ionizing Radiation Induces Heart and Cardiomyocyte Senescence

**Figure 1:**
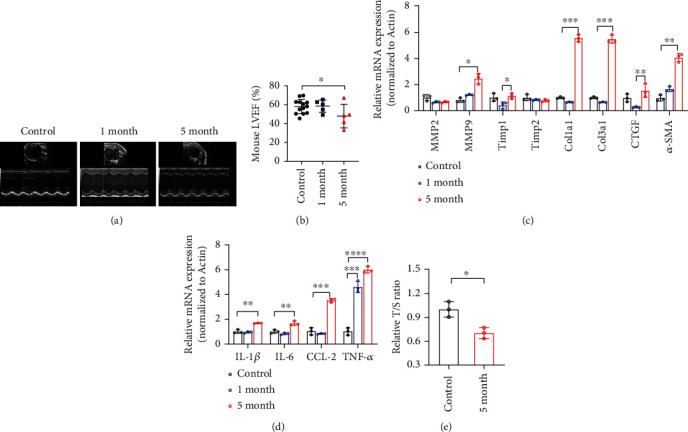
Ionizing radiation causes cardiac senescence. (a, b) Echocardiograph images and LVEF of mouse hearts in the control mice (control group) and in mice 1 and 5 months (1-month and 5-month groups, respectively) after local heart irradiation at a dose of 16 Gy. (c) q-PCR analysis mRNA of fibrosis-associated factors in the 1-month, 5-month, and control groups (^∗^*p* < 0.05, ^∗∗^*p* < 0.01, ^∗∗∗^*p* < 0.001; NS: not significant). (d) q-PCR analysis of the mRNA levels of inflammation-associated factors in the 1-month, 5-month, and control groups (^∗^*p* < 0.05, ^∗∗^*p* < 0.01, ^∗∗∗^*p* < 0.001, NS: not significant). (e) q-PCR analysis of telomere length in cardiac tissue from 1 month to 5 months, and control mice (^∗^*p* < 0.05).

**Figure 2:**
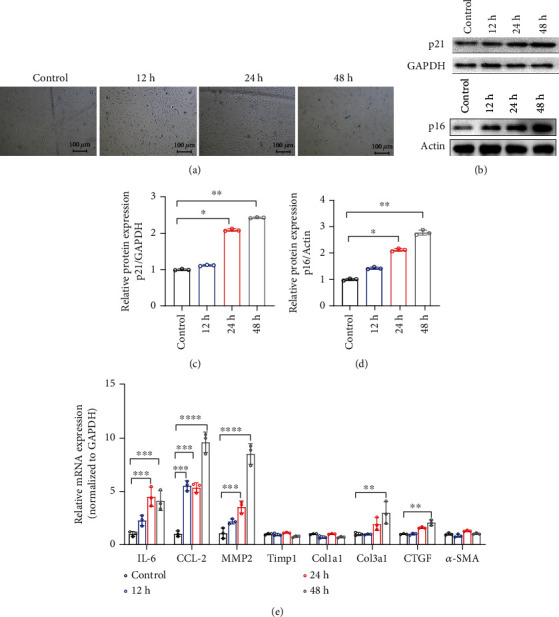
Ionizing radiation causes cardiomyocyte senescence. (a) Senescence-associated *β*-galactosidase (SA-*β*gal) staining of sham-irradiated and irradiated H9C2 cells. (b)–(d) Western blotting and quantification analyses of p21 and p16 protein expression in H9C2 cells in the control group and after 6h, 12h, and 24h of irradiation (^∗^*p* < 0.05, ^∗∗^*p* < 0.01). (e) q-PCR analysis of the mRNA levels of inflammation- and fibrosis-associated factors in H9C2 cells of the control, 6h, 12h, and 24h irradiation groups (^∗^*p* < 0.05, ^∗∗^*p* < 0.01, ^∗∗∗^*p* < 0.001, ^∗∗∗∗^*p* < 0.0001). Bar: 100*μ*m; h: hour.

IR can elicit cellular senescence in cardiomyocytes, fibroblasts, and epithelial cells [44]. Cardiac senescence always exhibits ventricular remodeling, telomere attrition, proinflammatory and profibrotic molecule secretion, and activation of the p53/p21 and/or p16 signaling pathways [45]. Our previous study showed that p21^−/−^ mice were more prone to severe RIHD after irradiation than wild-type mice [40]. In this study, we systematically investigated IR-induced cardiac senescence and detected cardiac senescence that manifested after 5 months. We then investigated whether radiation induced cardiac senescence phenotypes. Echocardiography results indicated enlarged cardiac chambers, thinner ventricular walls, and decreased left ventricular ejection fraction (Figures 1(a) and 1(b)). SASP levels in heart tissue detected by q-PCR showed that fibrosis-related factors, such as MMP9, Trimp1, Col1a1, Col3a1, CTGF, and *α*-SMA, and the inflammatory factors IL-1, IL-6, CCL-2, and TNF-*α* dramatically increased after 5 months of radiation (Figures 1(c) and 1(d)). Moreover, mice in the irradiated group had a shortened telomere length compared to control mice (Figure 1(e)). IR increased *β*-galactosidase staining in H9C2 cells at 12, 24, and 48 h (Figure 2(a)). The expression levels of the senescence-related proteins p21 and p16 were elevated after IR at 10 Gy. Furthermore, SASP factors such as IL-6, CCL-2, MMP-2, col3a1, and CTGF increased (Figure 2(e)). The DNA damage-associated repair protein *γ-*H2AX was overexpressed after IR (Figure S1). These findings indicate that radiation induces cardiac and cardiomyocyte senescence both in vivo and in vitro.

### 3.2. Ionizing Radiation Induces Lysine Residue Hyperacetylation and Modification of Cardiac Metabolic Enzymes

**Figure 3:**
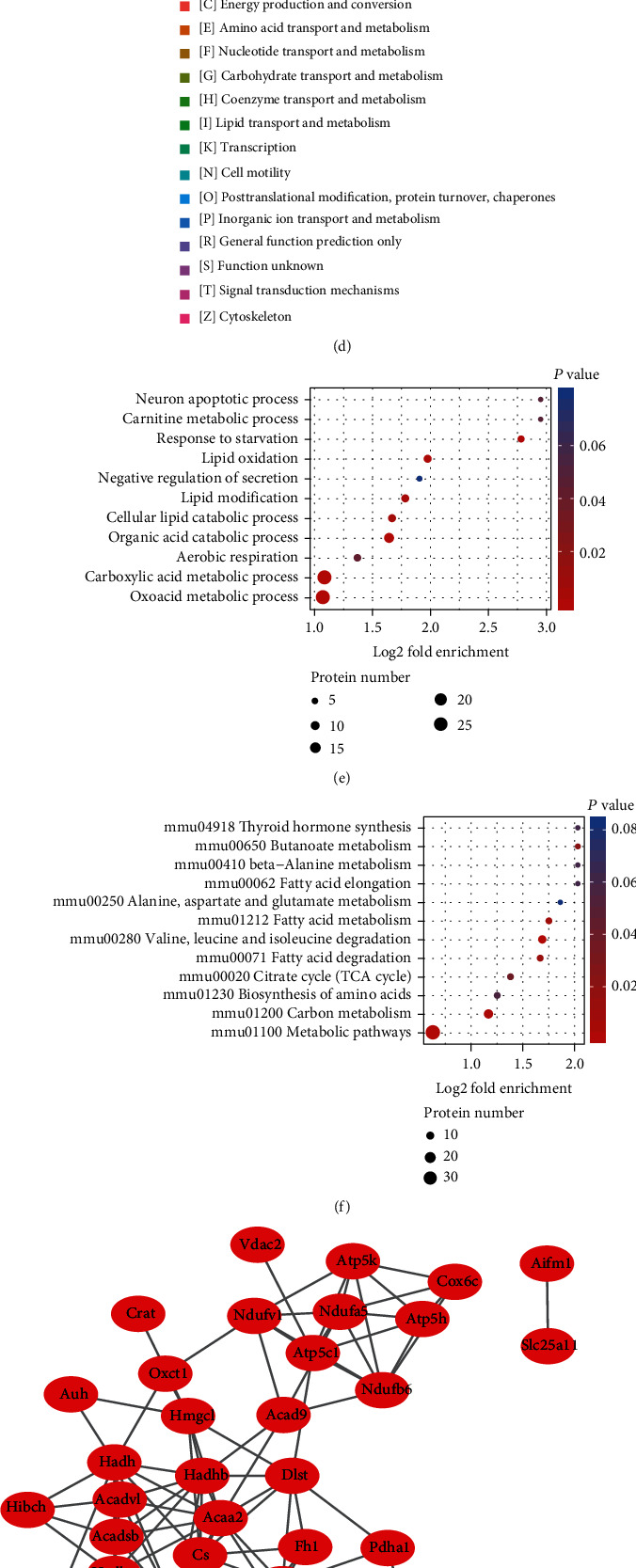
Ionizing radiation induces lysine residue hyperacetylation, thus modifying cardiac metabolic enzymes. (a) Radiation induces protein acetylation in mouse hearts after 5 months. (b) Radiation induces protein acetylation in H9C2 cells at different time points. (c) Experimental flow chart of proteomic analysis. (d) Clusters of Orthologous Groups/KOG functional classification chart of proteins corresponding to differentially expressed modification sites. (e) GO enrichment bubble plot of proteins corresponding to differentially expressed modification sites in the biological process category. (f) KEGG pathway enrichment bubble plot of proteins corresponding to differentially expressed modification sites. (g) PPI network of the DEPs. Red nodes represent upregulated proteins, and blue nodes represent downregulated proteins. (h) The CytoHubba of Degree method were used to extract the top 10 hub proteins from the PPI network. (i) The CytoHubba of DMNC method was used to extract the top 10 hub proteins from the PPI network.

Our previous study showed that irradiated heart tissue exhibited significant dysregulation of mitochondrial damage and metabolites, which could be characterized by inner mitochondrial membrane damage and decreased ATP synthesis [38]. To investigate the mechanism of acyl modifications in RIHD, we examined acetylation, succinylation, crotonylation, 2-hydroxyisobutyrylation, malonylation, and ubiquitination levels in sham- and 5-month-irradiated heart tissues. Compared to those in sham-irradiated mice, the levels of acetylation, crotonylation, 2-hydroxyisobutyrylation, and malonylation increased in the hearts of irradiated mice. Among these modifications, acetylation exhibited the most obvious upregulation (Figure S2). Posttranslational protein acetylation is involved in the regulation of metabolism [46]. To investigate whether IR could drive lysine acetylation changes, we measured the acetylation levels of irradiated heart tissue and H9C2 cells, and the results indicated that high-energy radiation could alter the acetylation status (Figures 3(a) and 3(b)).

Lysine acetylation proteomic analysis of the heart was conducted for the irradiated and control groups (Figure 3(c)). A total of 721 proteins with 2138 acetylation sites were discovered in the acetylation modification proteome data, of which 666 proteins with 1985 acetylation sites were identified (Supplementary Table 4). Sixty-one proteins with 80 acetylation sites were upregulated, and nine proteins with 10 acetylation sites were downregulated (Figure S3A–B).

This result is in accordance with the high-throughput acetyl-proteomic classification and enrichment analysis. Nearly half of the upregulated proteins and acetylation sites were located in the mitochondria (Figure S3C). Clusters of Orthologous Groups analysis was performed, and the results revealed that DEPs in acetylation sites were mainly clustered in energy production and conversion, lipid transport and metabolism, and amino acid transport and metabolism (Figure 3(d)). In addition, GO functional enrichment analysis revealed that most DEPs of the modification sites were enriched in single-organism processes, cellular processes, and metabolic processes in the biological process category (Figure 3(e)). Within the cellular component category, a large number of DEPs were categorized into mitochondrial categories (Figure S3D). Metabolic enzyme activity and binding were also enriched in the main molecular function category (Figure S3E). According to the KEGG database, differentially expressed acetylated proteins were enriched in different metabolic pathways, such as butanoate, beta-alanine, amino acid, and fatty acid metabolism (Figure 3(f)).

Sixty-nine proteins were filtered into the DEP PPI network complex using STRING, and the resulting PPI network contained 52 nodes and 129 edges (Figure 3(g)). The top 10 hub genes selected using the DMNC and degree methods (score ≥ 5,000) and node degree (score ≥ 10) in the CytoHubba plug-in included Atp5c1 (Atp5f1c) and HSP901b (Figures 3(h) and 3(i)).

### 3.3. Hyperacetylated Atp5f1c K55 Site Induces Metabolic Dysfunction and Cardiomyocyte Senescence

**Figure 4:**
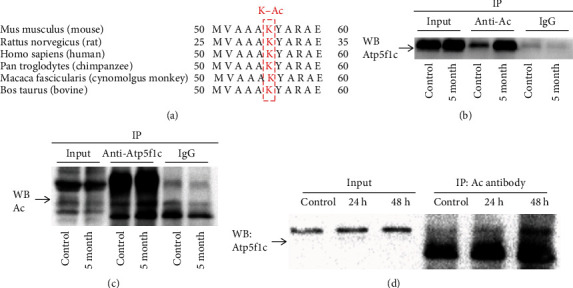
Ionizing radiation induces Atp5f1c acetylation in irradiated heart and cardiomyocytes. (a) Sequence alignment of mouse, rat, human, chimpanzee, monkey, and bovine Atp5f1c proteins. (b, c) Ionizing radiation induced Atp5f1c acetylation in heart tissue, as verified by Co-IP. (d) Ionizing radiation induced Atp5f1c acetylation in cardiomyocytes, as verified by Co-IP. K-Ac: lysine (K) acetylation; Co-IP: coimmunoprecipitation; WB: western blotting.

In the above acetyl-proteomic results, DEPs of acetylation modification related to fatty acid metabolism and energy metabolism were obviously overexpressed and extensively distributed in the mitochondria and mitochondrial membrane. In addition, we observed radiation-induced cardiac damage and ATP depletion. Among the highly acetylated proteins, Atp5f1c is mainly related to energy metabolism [47]. Other mitochondrial membrane ATP synthase subunits were also hyperacetylated after radiation, in accordance with the acetyl proteomic analysis results, including Atp5h and Atp5me (Atp5k), which produce ATP from ADP in the presence of a proton gradient across the membrane that is generated by electron transport complexes of the respiratory chain. Atp5f1c was the most hyperacetylated protein among ATP synthase subunits (Table 1). The lysine 55 site of Atp5f1c (Atp5f1c K55) was fairly conserved among mammalian species, as validated by the ClustalX 2.1 software, indicating that it has high fidelity (Figure 4(a)). The level of lysine acetylation after IR was confirmed by Co-IP in vivo and in vitro (Figures 4(b)–4(d)).

**Figure 5:**
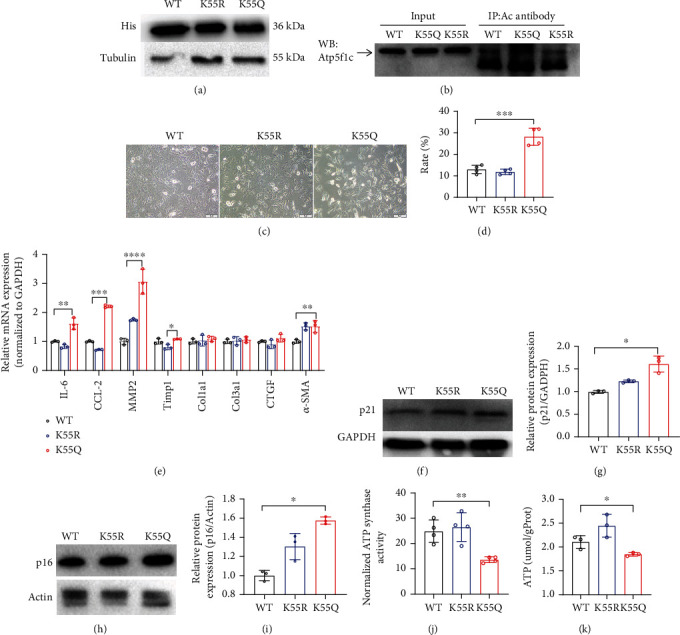
Atp5f1c is hyperacetylated at the 55th lysine site, which leads to metabolic disorders and senescence. (a) The expression of anti-His antibody after His-tagged Atp5f1c WT, K55R, and K55Q point mutation plasmids transfection in H9C2 cells. (b) Atp5f1c acetylation level verified by Co-IP after transfection of Atp5f1c WT, K55R, and K55Q point mutation plasmids in H9C2 cells. (c, d) Senescence-associated *β*-galactosidase (SABG) staining and statistical analysis after transfection of Atp5f1c WT, K55R, and K55Q point mutation plasmids in H9C2 cells (^∗^*p* < 0.05, ^∗∗^*p* < 0.01, ^∗∗∗^*p* < 0.001; NS: not significant). (e) q-PCR analysis of the mRNA levels of inflammation- and fibrosis-associated factors after transfection of Atp5f1c WT, K55R, and K55Q point mutation plasmids in H9C2 cells (^∗^*p* < 0.05, ^∗∗^*p* < 0.01, ^∗∗∗^*p* < 0.001, ^∗∗∗∗^*p* < 0.0001). (f)–(i) Western blotting and quantification analyses of p21 and p16 protein expression after transfection of Atp5f1c WT, K55R, and K55Q point mutation plasmids in H9C2 cells; relative protein levels were normalized to GAPDH or actin expression (^∗^*p* < 0.05). (j, k) ATP synthase activity and ATP production after transfection of Atp5f1c WT, K55R, and K55Q plasmids into H9C2 cells (^∗^*p* < 0.05, ^∗∗^*p* < 0.01, ^∗∗∗^*p* < 0.001; NS: not significant). Co-IP: Coimmunoprecipitation; WB: western blotting; WT: wild type.

As Atp5f1c is an enzyme central to metabolism, radiation could cause hyperacetylation of cardiac Atp5f1c. Moreover, considering that Atp5f1c K55 has fidelity in various mammals, we further investigated whether the alterations in Atp5f1c K55 acetylation mediated the cardiac senescence and energy metabolism caused by radiation. Subsequently, we constructed purified His-tagged proteins containing a mutant site of Atp5f1c K55 to Gln (mimic acetyl-modification, K55Q) and Arg (mimic deacetyl-modification, K55R) in H9C2 cells. The expression of the tag protein showed that the plasmid was successfully transfected into H9C2 cells (Figure 5(a)). Atp5f1c K55 acetylation was further verified by Co-IP in H9C2 cells transfected with different plasmids (Figure 5(b)). Atp5f1c K55 hyperacetylation increased senescent cells in the *β*-galactosidase strain; promoted cytokine IL-6, CCL-2, MMP2, and *α*-SMA secretion; and overexpressed senescence-associated proteins p21 and p16 (Figures 5(c)–5(i)). In addition, the hyperacetylated Atp5f1c K-55 site exhibited decreased enzymatic activity and ATP synthesis (Figures 5(j) and 5(k)). These findings suggest that K55 acetylation of Atp5f1c plays a significant role in cellular senescence and energy metabolism.

### 3.4. Sirt4 and Sirt5 Mediate Atp5f1c K55-Ac Deacetylation

**Figure 6:**
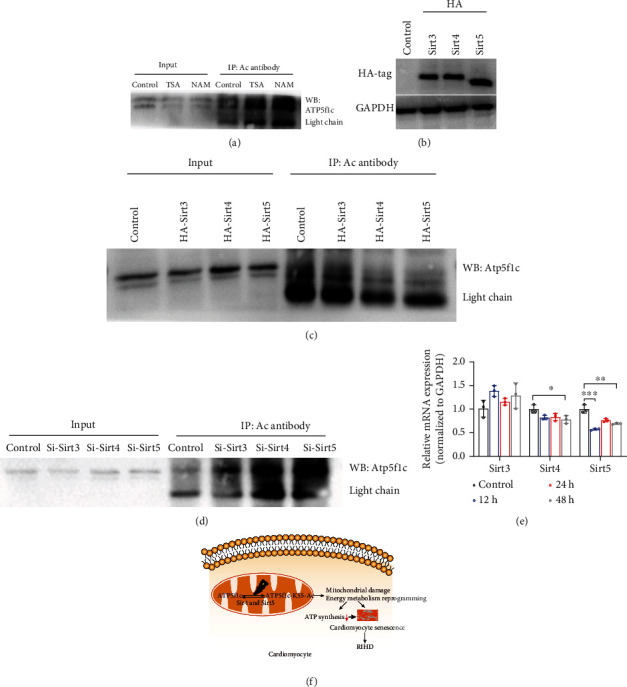
Sirt4 and Sirt5 mediated Atp5f1c K55 deacetylation. (a) Effect of trichostatin A (TSA) and nicotinamide (NAM) on Atp5f1c acetylation levels in H9C2 cells. (b) The expression of HA-tag after HA-tagged Sirt3, Sirt4, and Sirt5 plasmids was transfected into H9C2 cells. (c) Atp5f1c acetylation verified by Co-IP after HA-tagged Sirt3, Sirt4, and Sirt5 plasmids was transfected into H9C2 cells. (d) Atp5f1c acetylation level verified by Co-IP after siRNA of Sirt3, Sirt 4, and Sirt 5 was transfected into H9C2 cells. (e) q-PCR analysis of the mRNA levels of Sirt3, Sirt4, and Sirt 5 in H9C2 cells of the control, 6, 12, and 24 h irradiation groups (^∗^*p* < 0.05, ^∗∗^*p* < 0.01, ^∗∗∗^*p* < 0.001). (f) Schematic diagram of the study design. WB: western blotting; h: hour; WB: western blotting; RIHD: radiation-induce heart damage.

The above experiments demonstrated that Atp5f1c K55 acetylation (Atp5f1c K55-AC) influences ATP synthesis by ATP synthase enzyme activity and promotes cardiomyocyte senescence. Acetylation of nonhistone proteins is regulated by the classical HDAC family and the NAD^+^-dependent Sirt family. We then investigated the effects of the Sirt inhibitor nicotinamide and the HDAC inhibitor trichostatin A on Atp5f1c acetylation [48]. As nicotinamide treatment clearly increased Atp5f1c acetylation (Figure 6(a)), and NAD^+^-dependent Sirts were involved in Atp5f1c acetylation. Considering that Atp5f1c is localized in the mitochondria, the major mitochondrial deacetylases Sirt3, Sirt4, and Sirt5 were selected for further investigation. H9C2 cells were transfected with empty vector and Sirt3, Sirt4, and Sirt5 overexpression plasmids with an HA-tag. The presence of HA-tag was used to demonstrate the expression of Sirt plasmids (Figure 6(b)). The decreased level of Atp5f1c acetylation was validated using coimmunoprecipitation (Figure 6(c)). In parallel, siRNA of Sirt3, Sirt4, and Sirt5 was also transfected into H9C2 cells, and the efficiency of siRNA was confirmed by qPCR (Figure S4). Co-IP results showed that downregulation of Sirt4 and Sirt5 upregulated Atp5f1c acetylation levels (Figure 6(d)). The gene expression of Sirt4 and Sirt5 decreased after IR, and the decrease in Sirt5 was more pronounced. The above results indicate that IR-induced Atp5f1c acetylation is mediated by Sirt4 and Sirt5.

## 4. Discussion

Lysine acetylation-mediated metabolic regulation is involved in cardiac senescence. In this study, we first reported that IR upregulates Atp5f1c K55-Ac, affects ATP synthase activity, and promotes impairment of energy metabolism and cardiomyocyte senescence. The present study provides novel insights into the mechanisms of RIHD. DNA damage-induced double-strand breaks have been recognized as a significant factor in senescence [49]. IR led to cellular senescence, as indicated by decreased cardiac function, shortened telomeres, *β*-galactosidase staining, senescence-related proteins p21 and p16, and SASP, such as MMP9, Trimp1, Col1a1, Col3a1, CTGF, *α*-SMA, IL-1*β*, IL-6, CCL-2, and TNF-*α*. These cytokines are also associated with inflammation and fibrosis. Irradiated endothelial cells cause damage to adjacent cells through SASP regulation [50]. RIHD arises from cell apoptosis and inflammatory and fibrotic processes, which can lead to the thickening of blood vessels and fibrotic scar tissue in the heart [15]. Several studies have linked altered lysine acetylation to the development of radiation-induced damage [51]. In this study, acetylated protein levels were extensively elevated in irradiated heart tissue and H9C2 cells, compared to those in the control groups. Subsequent subcellular localization and functional enrichment analyses via acetyl-proteomics indicated that DEPs at different acetylated sites were enriched in mitochondrial and energy metabolism. Interestingly, we previously discovered that irradiated heart tissue exhibited significant dysregulation of mitochondrial damage and metabolites [38]. In addition to the myocardium, radiation-induced mitochondrial damage in the skeletal muscle alters the proteins involved in energy metabolism-related processes [52].

Atp5f1c is a component of an ATP synthase complex located in the mitochondrial inner membrane that produces ATP from ADP in the presence of a proton gradient across the membrane generated by electron transport complexes of the respiratory chain [47]. It was previously indicated that bedaquiline, an FDA-approved drug that can silence the Atpf1c expression in vitro by targeting Atp5f1c, can inhibit mitochondrial ATP production [53]. Moreover, mitochondrial ATP synthase may be a potential drug target against aging, according to a study on the activation of the typical longevity AMPK/mTOR pathway in Alzheimer's disease [54]. Atp5f1c is an important enzyme involved in metabolic pathways. In this study, IR significantly increased Atp5flc K55 acetylation levels and Atp5f1c K55-Ac overexpression, which induce metabolic disorder.

Acetylation levels of mitochondrial metabolic enzymes are affected by NAD^+^-dependent deacetylase sirtuins (Sirt) [55]. Sirt is an NAD^+^-dependent deacetylase and ADP-ribosyltransferase related to energy metabolism and senescence [56, 57]. Sirt1 is the most extensively studied protein and is mainly located in the cytoplasm and nucleus [58]. Sirt1 regulates oxidative stress by deacetylating Pgc-1*α* and contributing to Nrf2 transcription in chromium-induced lung injury [59]. In addition, Sirt3, Sirt4, and Sirt5 are mainly located in the mitochondria and are involved in metabolic reactions and antioxidant properties [60]. The ratio of NAD^+^ to NADH, which is closely related to glycolysis and the tricarboxylic (TCA) cycle, has been investigated in cellular and mitochondrial metabolism [61]. NAD^+^ levels in mammalian cells and tissues decline with age [62]. Loss of function of the NAD^+^-dependent enzyme Sirt has been linked to aging-related diseases, such as cancer, insulin resistance, heart disease, fibrosis, and neurodegeneration [63].

ATP synthase hyperacetylation can affect cardiac energy metabolism and is regulated by Sirt3 in heart failure [64]. Label-free quantitative proteomics of mouse liver mitochondria indicated that the absence of Sirt3 regulated the acetylation of multiple metabolism-related proteins [65]. Loss of mitochondrial Sirt function, especially Sirt3, has been linked to several age-related pathologies including cancer, insulin resistance, heart disease, fibrosis, and neurodegeneration [63]. In general, Sirt3 is regarded as a major deacetylase in the mitochondria [66]. However, in our study, the overexpression of both Sirt4 and Sirt5 decreased the acetylation level of Atp5flc. Protein activity is regulated through deacetylation of lysine residues. Sirt4 also has an important role in insulin secretion, fatty acid oxidation, amino acid metabolism, ATP homeostasis, and cardiovascular diseases [56]. Sirt4 deacetylates malonyl-CoA decarboxylase and decreases enzyme activity, limiting fatty acid oxidation under adequate nutrition conditions [67]. Guo et al. suggested that Sirt4 dramatically deacetylated the MTP*α* K350-Ac, K383-Ac, and K406-Ac sites, and that MTP*α* acetylation plays an important role in lipid catabolism in nonalcoholic fatty liver disease [68]. Similar to Sirt4, Sirt5 also plays an important role in metabolic adaptations. Sirt5 appeared to regulate the heart function. Sirt5 KO mice developed hypertrophic cardiomyopathy and showed reduced cardiac function during aging. Although Sirt3, Sirt4, and Sirt5 are mainly localized in the mitochondrial matrix [61], inhibition of Sirt4 increases fat oxidative capacity and mitochondrial function in liver and muscle cells [69], whereas the liver of Sirt3^−/−^ mice shows decreased *β*-oxidation of fatty acids [70]. In this study, we found that the expression of Sirt4 and Sirt5 decreased after IR, and that Sirt4- and Sirt5-mediated Atp5f1c acetylation promoted cardiomyocyte senescence.

## 5. Conclusion

In this study, we revealed that IR induced cardiomyocyte senescence and regulated the acetylation level of Atp5f1c, a key enzyme in energy metabolism, providing novel insights into radiation-induced senescence mediated by metabolic regulation. Atp5f1c K55-Ac in radiation-induced cells led to cardiomyocyte ATP production and cell senescence, and Sirt4 and Sirt5 were found to mediate Atp5f1c deacetylation. This provides a sufficient theoretical basis for elucidating the pathogenic mechanisms of radiation-induced heart disease and identifying potential therapeutic targets.

## Figures and Tables

**Table 1:** Modified K sites of ATP synthase subunit in radiated heart by proteomic profiling of lysine acetylation.

Protein accession	Position	Ratio	Regulated type	*p* value	Amino acid	Gene name
Q91VR2	55	1.346	Up	0.00080	K	Atp5f1c
Q9DCX2	95	1.202	Up	0.048	K	Atp5h
Q06185	34	1.308	Up	0.025	K	Atp5me

## Data Availability

The data used to support the finding of this study are available from the corresponding author upon request.

## Abbreviations

IR: Ionizing radiation
RIHD: Radiation-induced heart damage
Co-IP: Coimmunoprecipitation
SASP: Senescence-associated secretory phenotype
SSD: Source-to-surface distance
TMT: Tandem mass tags
NADH: Nicotinamide adenine dinucleotide hydrogen
NAD^+^: Nicotinamide adenine dinucleotide
DEPs: Differentially expressed proteins
KEGG: Kyoto Encyclopedia of Genes and Genomes
LV: Left ventricle
EF: Ejection fraction.

## Supplementary Materials

Supplementary Materials
*Supplementary Material:* Figure S1: immunofluorescence labeling of *γ*HAX in sham-irradiated and each irradiated H9C2 cell. Figure S2: acyl modifications of heart tissue after ionizing radiation (a)–(f). Acetylation, succinylation, crotonylation, 2-hydroxyisobutyrylation, malonylation, and ubiquitination level of sham- and 5-month-irradiated group. Figure S3: (a) volcano plot of differentially expressed modification sites. (b) Histogram of the number distribution of differentially expressed proteins and modification sites in different comparison groups. (c) Subcellular localization chart of proteins corresponding to differentially expressed modification sites. (d) GO (Gene Ontology) enrichment bubble plot of proteins corresponding to differentially expressed modification sites in three categories (e). Cellular component (f). Molecular function. Figure S4: q-PCR validation results of Sirt3, Sirt4, and Sirt5 siRNA. Data S1: search method of database. *Supplementary Table:* Supplementary Table 1: the primers of Atp5f1c-6his, Atp5f1c K55R-6his, and Atp5f1c K55Q-6his mutant plasmids. Supplementary Table 2: the primers of Sirt3, Sirt4, and Sirt5 overexpression plasmids. Supplementary Table 3: oligonucleotide sequences of siRNA. Supplementary Table 4: mouse primer sequences used for q-PCR Supplementary Table 5: rat primer sequences used for q-PCR. Supplementary Table 6: basic statistical table of MS results.
