# Supplementary material for: Acetylation of Atp5f1c Mediates Cardiomyocyte Senescence via Metabolic Dysfunction in Radiation-Induced Heart Damage
Source: Oxid Med Cell Longev. 2022 Sep 15;2022:4155565. doi: 10.1155/2022/4155565 (PMC9499811; doi:10.1155/2022/4155565)
Supplement: Supplementary Materials — Supplementary Material: Figure S1: immunofluorescence labeling of γHAX in sham-irradiated and each irradiated H9C2 cell. Figure S2: acyl modifications of heart tissue after ionizing radiation (a)–(f). Acetylation, succinylation, crotonylation, 2-hydroxyisobutyrylation, malonylation, and ubiquitination level of sham- and 5-month-irradiated group. Figure S3: (a) volcano plot of differentially expressed modification sites. (b) Histogram of the number distribution of differentially expressed proteins and modification sites in different comparison groups. (c) Subcellular localization chart of proteins corresponding to differentially expressed modification sites. (d) GO (Gene Ontology) enrichment bubble plot of proteins corresponding to differentially expressed modification sites in three categories (e). Cellular component (f). Molecular function. Figure S4: q-PCR validation results of Sirt3, Sirt4, and Sirt5 siRNA. Data S1: search method of database. Supplementary Table: Supplementary Table 1: the primers of Atp5f1c-6his, Atp5f1c K55R-6his, and Atp5f1c K55Q-6his mutant plasmids. Supplementary Table 2: the primers of Sirt3, Sirt4, and Sirt5 overexpression plasmids. Supplementary Table 3: oligonucleotide sequences of siRNA. Supplementary Table 4: mouse primer sequences used for q-PCR Supplementary Table 5: rat primer sequences used for q-PCR. Supplementary Table 6: basic statistical table of MS results. [file 4155565.f1.zip › revisedSupplementary Material.docx]

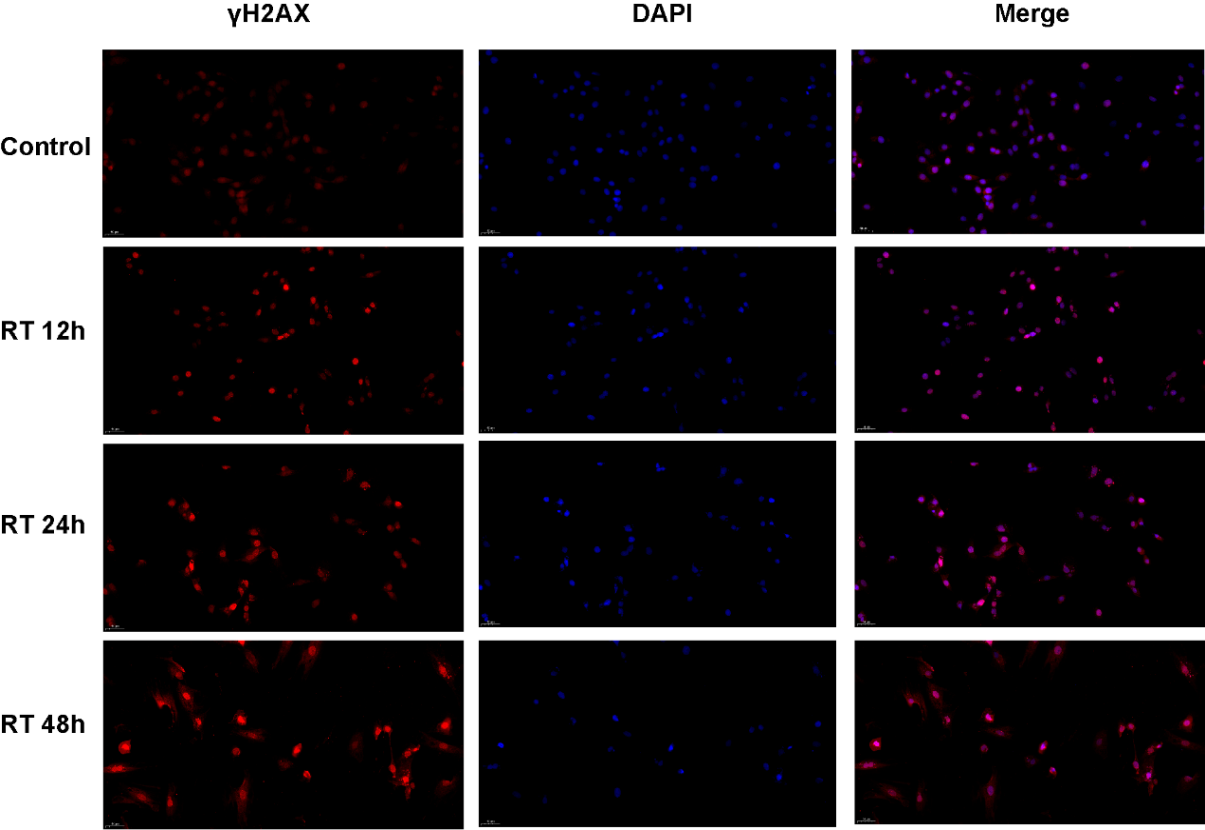


Figure S1. Immunofluorescence labeling of γHAX in sham-irradiated and each irradiated H9C2 cell.


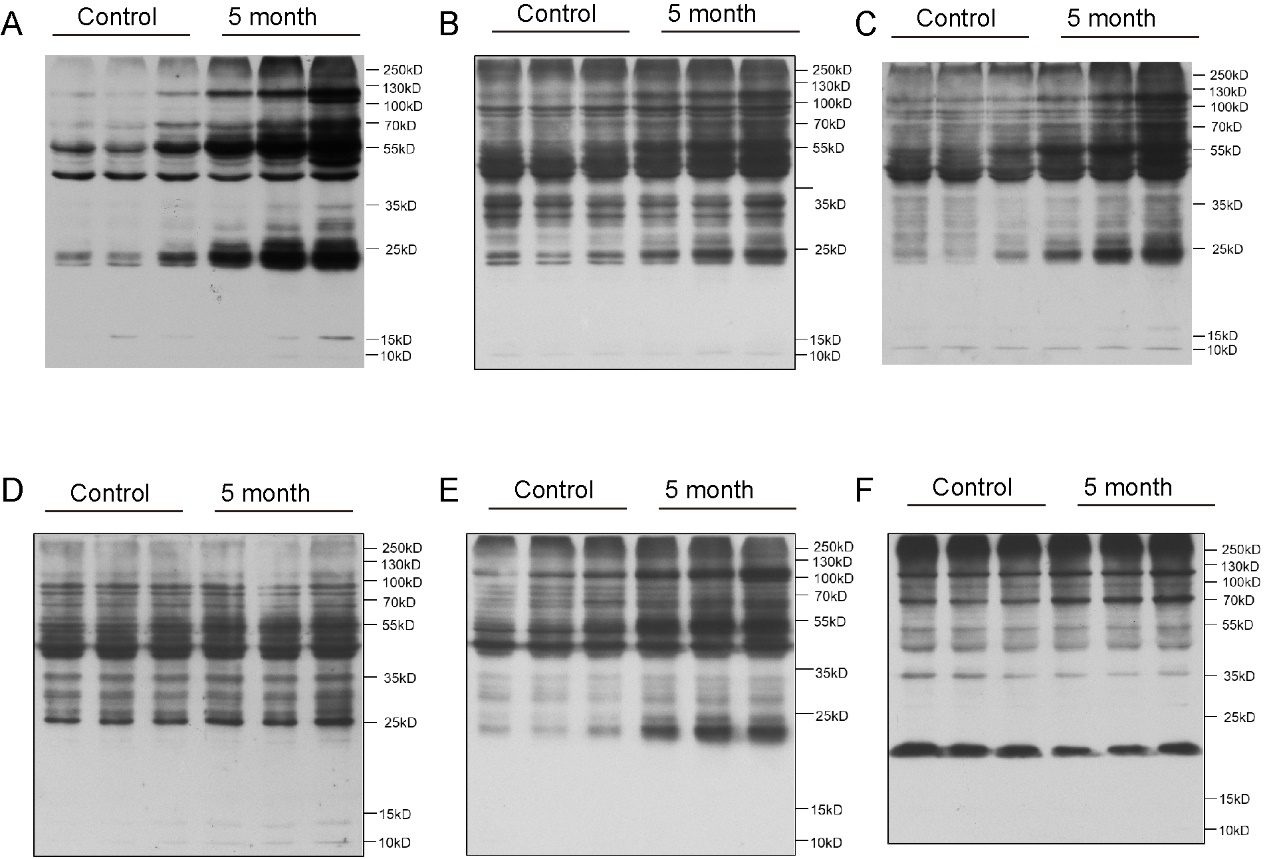


Fig S2. Acyl modifications of heart tissue after ionizing radiation.

A-F. acetylation, succinylation, crotonylation, 2-hydroxyisobutyrylation, malonylation and ubiquitination level of sham- and 5-month irradiated group.


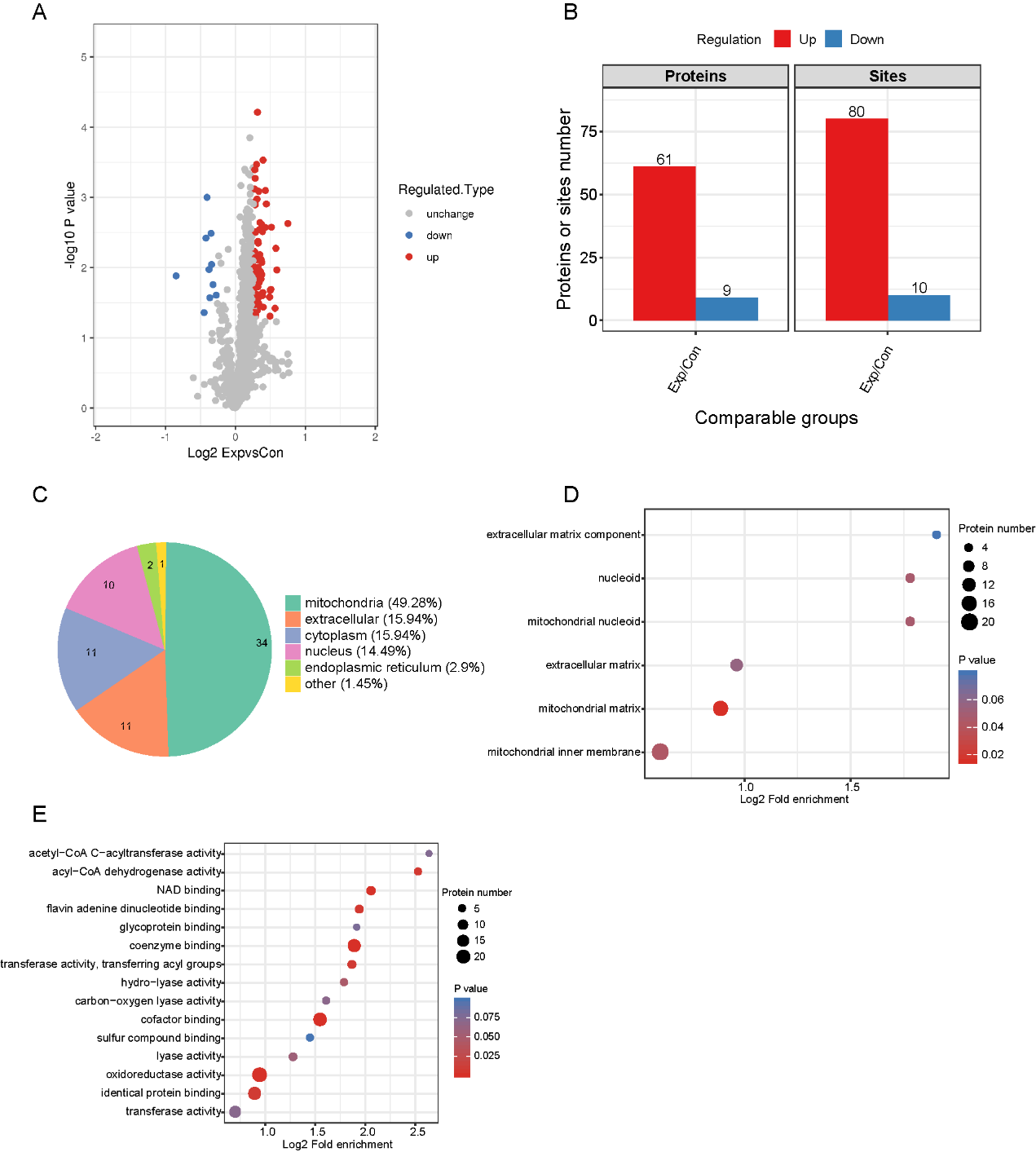


Figure S3. (A) Volcano plot of differentially expressed modification sites. (B)Histogram of the number distribution of differentially expressed proteins and modification sites in different comparison groups. (C) Subcellular localization chart of proteins corresponding to differentially expressed modification sites. (D) GO (Gene Ontology) enrichment bubble plot of proteins corresponding to differentially expressed modification sites in three categories (E). Cellular Component; (F). Molecular Function.


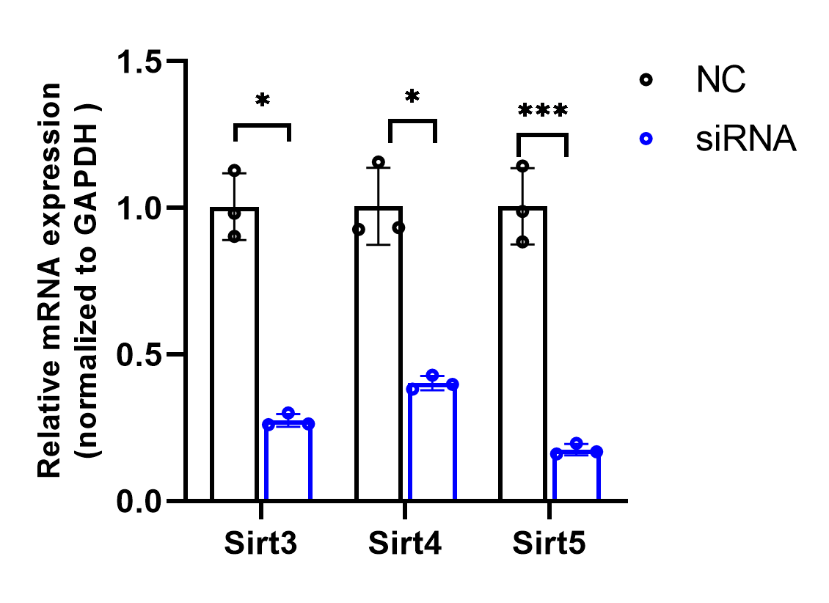


Figure S4: q-PCR validation results of Sirt3, Sirt4 and Sirt5 siRNA.

**Data S1. Search method of database**

Tandem mass spectra were searched against UniProt database concatenated with reverse decoy database. Trypsin/P was specified as cleavage enzyme allowing up to 4 missing cleavages. The mass tolerance for precursor ions was set as 20 ppm in First search and 5 ppm in Main search, and the mass tolerance for fragment ions was set as 0.02 Da. Carbamidomethyl on Cys was specified as fixed modification and acetylated modification and oxidation on Met were specified as variable modifications. FDR was adjusted to < 1% and minimum score for modified peptides was set > 40.
